# Supplementary material for: Inferring the internal structure of groups through the integration of statistical learning and causal reasoning
Source: Nat Commun. 2026 Jan 23;17:1959. doi: 10.1038/s41467-026-68754-0 (PMC12929721; doi:10.1038/s41467-026-68754-0)
Supplement: Supplementary file 1 — Supplementary Information [file 41467_2026_68754_MOESM1_ESM.pdf]

Inferring the internal structure of groups through the integration of statistical learning and causal reasoning: supplementary information

1 Supplementary methods

1.1 Model specifications

Let $A = \{a_1, \ldots, a_k\}$ be a set of agents, $R = \{r_1, \ldots, r_m\}$ be a set of social relations, and $T = \{t_1, \ldots, t_m\}$ be a set of social interaction types (one for each social relation). We define a *social structure* $S = \{C, E\}$ over the agents in A as

- An assignment of agents to clusters, so that $C(a)$ is the cluster assignment of agent a, and
- a set $E \subset C \times C \times R$ of typed edges between clusters, so that $E(c_1, c_2)$ is the type of relation between clusters c_1 and c_2 (or ϕ if there is no edge between them). For symmetric relations (e.g.: friendship), $E(c_1, c_2) = E(c_2, c_1)$ for any pair of clusters.

We define a social interaction $d = \{t, \text{init}, \text{recip}, \text{response}\}$, in terms of its interaction type $t \in T$, the initiating agent $\text{init} \in A$, the recipient $\text{recip} \neq \text{init}$, the recipient's response $\text{response} \in \{\text{yes}, \text{no}\}$. Given a sequence $D = \{d_1, \ldots, d_n\}$ of interactions

between agents in A, our model computes a probability distribution $P(S|D)$ over possible social structures via Bayes' rule:

$$P(S|D) \propto P(D|S)P(S) \tag{1}$$

To compute $P(S)$, we first use a Chinese Restaurant Process with concentration parameter equal to 3 to define a prior distribution $P(\pi)$ over all possible partitions of agents into groups. Then, for each partition π, and each pair of groups within π, we assume an edge exists between these groups with probability .5. We then restrict the resulting prior distribution to only include acyclic structures. To compute the likelihood $P(D|S)$, we assume that interactions are conditionally independent given the underlying structure, so that $P(D|S)$ factors as

$$P(D|S) = \prod_{i=1}^{n} P(d_i|S) \tag{2}$$

Each $P(d_i|S)$ is computed according to

$$P(d_i|S) = P(\text{init}|S,t)P(\text{recip}|S,t,\text{init})P(\text{response}|S,t,\text{init},\text{recip}) \tag{3}$$

where t is the interaction type of d_i. The three terms on the right hand side of equation (3) are given by the *naive sociology model* for interactions of type t.

1.2 Naive sociologies

Each naive sociology module encodes a set of expectations governing how a particular type of social relation influences the likelihood of a particular type of social interaction. For this project, we defined naive sociologies for three relation/interaction type pairs: authority/orders, friendship/invitations, mentorship/advice requests. The details and

free parameters of each module are shown below.

Authority/orders:

- For orders, we define $P(\text{init} = a|S)$ as proportional to the total number of subordinates under agent a, counting both direct reports and subordinates of direct reports, though the latter are included at a discounted rate. To compute this, let $dist(c_1, c_2)$ be the length of the shortest directed path from cluster c_1 to cluster c_2 containing only authority-typed edges (or ∞ if no such path exists).[1] We then define $subs(a)$ as

$$\sum_{c \neq C(a)} |c| * \beta_{down}^{dist(C(a),c)-1} \tag{4}$$

where $|c|$ is the number of agents in cluster c, and $\beta_{down} \in (0, 1)$ is a discount parameter. Intuitively, this captures the expectation that agents with more subordinates are more likely to give orders, but distant subordinates may carry less weight than direct subordinates. We then define $P(\text{init} = a|S) = subs(a)/Z$, where Z is a normalizing factor equal to $\sum_{a' \in A} subs(a)$.

- Given the initiating agent a, we define $P(\text{recip} = b|S, \text{init} = a)$ in terms of the directed distance between a and b in the hierarchy. To this end, we extend the distance function $dist(a, b)$ so that if b is *above* a in the hierarchy, then $dist(a, b) = -dist(b, a)$. We then assign b a recipient score

$$recipScore(b) = \begin{cases} \beta_{down}^{dist(a,b)-1} & \text{if } dist(a,b) \geq 0 \\ \beta_{up}^{dist(a,b)-1} & \text{if } dist(a,b) < 0 \end{cases} \tag{5}$$

where $\beta_{down} \in (0, 1)$ is the same discount parameter used above, and $\beta_{up} > 1$ is a

[1]We use $dist(a, b)$ as shorthand for $dist(C(a), C(b))$ when there is no chance for confusion.

second discount parameter. Intuitively, this score function captures the intuitions that a is much more likely to give orders to subordinates than superiors, and a is more likely to give orders to agents closer to them in the hierarchy. Finally, we define $P(\text{recip} = b|S, \text{init} = a) = recipScore(b)/Z$, where $Z = \sum_{a' \in A} recipScore(a')$ is a normalizing factor.

- Given init $= a$ and recip $= b$, we define the probability of b responding positively as

$$P(\text{response} = \text{yes}|S, \text{init} = a, \text{recip} = b) = \begin{cases} \beta_{pos} & \text{if } dist(a, b) > 0 \\ 1 - \beta_{pos} & \text{if } dist(a, b) \leq 0 \end{cases} \tag{6}$$

  where $\beta_{pos} \in (.5, 1)$ is a parameter capturing the social cost of refusing an order from a superior.

Friendship/invitations:

- For invitations, we define $P(\text{init} = a|S)$ according to the size of agent a's friend group with diminishing marginal returns. Specifically, let

$$disc(n) = \begin{cases} 1 & \text{if } n = 1 \\ \beta_{init}^{n} + disc(n-1) & \text{if } n > 1 \end{cases} \tag{7}$$

  where $\beta_{init} \in (0, 1)$ is a discount factor. We then define $P(\text{init} = a|S) = disc(|C(a)| - 1)/Z$, where $|C(a)|$ is the number of agents in cluster $C(a)$, and $Z = \sum_{a' \in A} disc(|C(a')|)$ is a normalizing factor.

- Given init $= a$, we define $P(\text{recip} = b|S, \text{init} = a)$ as $\beta_{high} \in (0, 1)$ if $C(a) = C(b)$, and $\beta_{low} < \beta_{high}$ otherwise.

- We define $P(\text{response} = \text{yes}|S, \text{init} = a, \text{recip} = b)$ as β_{high} if $C(a) = C(b)$ and β_{low} otherwise.

Mentorship/advice requests:

- For advice requests, we define $P(\text{init} = a|S)$ to be highest for agents with mentors but no mentees, lowest for agents with mentees but no mentors, and an intermediate value for agents with both mentors and mentees. Specifically, we assign a score of 1 to agents with at least one mentor but no mentees, $\beta_{request} \in (0, 1)$ to agents with at least one mentor and at least one mentee, and $\beta^2_{request}$ for agents with at least one mentee and no mentors. We then define $P(\text{init} = a|S) = score(a)/Z$, where $Z = \sum_{a \in A} score(a)$ is a normalizing factor.

- Given $\text{init} = a$, we define $P(\text{recip} = b|S, \text{init} = a)$ in terms of a ranking of likely targets for seeking help, with the decrease in probability determined by a decay parameter $\beta_{recip} \in (0, 1)$. In particular, if b is a mentor of a, then we assign b a $recipScore(b) = 1$; if b and a share a mentor we then $recipScore(b) = \beta_{recip}$; otherwise $recipScore(b) = \beta^2_{recip}$. We then define $P(\text{recip} = b|S, \text{init} = a) = recipScore(b)/Z$, where Z is the usual normalizing factor.

- We define $P(\text{response} = \text{yes}|S, \text{init} = a, \text{recip} = b)$ to be β_{pos} if b is a mentor to a and $1 - \beta_{pos}$ otherwise.

1.3 Inference and prediction

There are three kinds of inferences and predictions that participants made in our experiments: the posterior probability of specific structures (Experiment 1), the likelihood of specific future interactions (Experiment 2), and the likelihood of one agent convincing another agent to make one of three types of decision. The posterior structure probability $P(S|D)$ is given directly by equation (1). For Experiment 2, we compute the probability of a specific interaction given the previous interactions, $P(d|D)$, by

marginalizing out the structure posterior, i.e.:

$$P(d|D) = \sum_{S} P(d|S)P(S|D) \tag{8}$$

where $P(S|D)$ is given by Equation (1). For Experiment 3 predictions, we ask participants about three types of choices that agents may be considering (working an extra shift, seeing a certain movie, taking an optional seminar), and our model assumes that an agent considering each type of choice would be most greatly influenced by agents with a particular relation to them (managers, friends, mentors, respectively). We therefore estimate the degree of influence that a has over b on choice type c to be the probability that a has the corresponding relation type to b, which we compute by marginalizing over all possible structures. I.e.:

$$Influence(a, b, c) = P(r(a, b) = c|D) = \sum_{S} \mathbb{I}[r(a, b) = c] * P(S|D) \tag{9}$$

where $r(a, b) = c$ specifies that the relation between a and b is the one corresponding to choice type c, and $\mathbb{I}$ is an indicator function.

1.4 Alternate models

In addition to our main model, we tested four alternate models. The first three alternate models were variations of the main model, except they assumed that the observer would use the same naive sociology to interpret all social data. Thus, these models were identical to the one described in the previous section, but instead of using the naive sociology appropriate for the observed interaction type, they used the same naive sociology for all observations. Other than this permutation, these alternate sociology models were defined and implemented exactly as the main model. In Experiments 2 and

3, we also tested a simple heuristic model that bases its inferences on observed interaction frequencies. For each possible interaction d, the frequency model computed the number of times that interaction occurred in a stimulus dataset D. For interactions that were not observed, it sampled a number from a uniform prior between 0 and 5. We then normalized these interaction counts by the total number of interactions (including the sampled values for unobserved interactions), and used these frequencies as estimates of $P(d'|D)$ for the target interactions participants were queried about.

1.5 Implementation

Each naive sociology module contains three free parameters. Given a set of parameter values, the module defines a function mapping a social structure S to a probability distribution $P(D|S)$ over social interactions. We estimated these parameter values through a set of unregistered pilot studies, one for each naive sociology, each with $n = 40$ participants with US-based IP addresses recruited through Prolific. In each study, participants read through a set of instructions explaining how to interpret diagrams of social structures. Participants then saw 8 trials. Each trial depicted a different social structure, then asked participants to rate a) the likelihood that each agent would initiate a particular type of interaction, b) given a particular initiating agent, the likelihood that they would interact with each other agent, and c) given the initiating and receiving agent, how the receiver is likely to respond. Participants used continuous sliders for all questions, ranging from 0 (definitely not this one) to 100 (definitely this one). We then averaged participant responses for each trial, yielding an estimated value for each stage of each type of interaction. We then used a Maximum Likelihood Estimation procedure the fit the free parameters of each sociology module to participant data, and used these parameter values to generate predictions across all three studies. The MLE parameter values are listed below:

1. Authority: $\beta_{down} = .27$, $\beta_{up} = 4.2$, $\beta_{pos} = .8$.

2. Friendship: $\beta_{init} = .5$, $\beta_{high} = .8$, $\beta_{low} = .2$.

3. Mentorship: $\beta_{request} = .5$, $\beta_{high} = .8$, $\beta_{low} = .2$.

To generate experimental predictions, we approximated the structure posterior $P(S|D)$ via a Metropolis-Hastings algorithm that iterated between resampling cluster assignments and resampling edges between clusters. Based on initial stability testing, we iterated this algorithm for 60,000 samples, after a burn-in period of 5,000 samples.

1.6 Experiments

1.6.1 Stimulus generation and trial selection

Each stimulus video depicted a sequence of 6-8 social interactions of a specific type. To select these stimulus sequences, we first generated, for each experiment, a large number of random interaction sequences via the following procedure:

1. Randomly sample a social structure S from a uniform prior.

2. Randomly sample a sequence of 8 interactions from the likelihood function $P(D|S)$ of the corresponding structure.

3. Check if the sequence meets the following criteria:

   - At least one and no more than two rejected interactions (i.e.: "no" responses)

   - No more than two repeats of any interaction

   - At most one agent who was not involved in any of the sampled interactions.

   If the sequence fails to meet any of these criteria, omit it from the list of candidates

4. Repeat until 10,000 sequences have been generated

We then selected 8 interaction sequences for each experiment based on criteria described further below. Once the interaction sequences were selected, we converted them into animated videos in Processing.[2]

Experiment 1

For Experiment 1, we first applied our main model to estimate $P(S|D)$ for each sequence, then chose the four structures $S_1, \ldots, S_4$ with the highest posterior probabilities, and renormalized the probabilities to obtained a truncated posterior for these four structures. We then defined four "probability profiles" and chose 2 trials for which the truncated posterior distribution met that profile. The profiles were:

- 1 highly likely structure ($P(S|D) > .5$) and three unlikely structures ($P(S|D) < .15$).
- 1 highly likely structure ($P(S|D) > .5$), 1 moderately likely structure ($P(S|D) > .35$), and 2 unlikely structures ($P(S|D) < .15$).
- 2 moderately likely structures ($P(S|D) > .4$) and 2 unlikely structures ($P(S|D) < .15$).
- Three moderately likely structures ($P(S|D) > .3$) and one unlikely structure ($P(S|D) < .1$).

1.6.2 Experiment 2

For Experiment 2, we augmented each interaction sequence with a randomly chosen "target" agent, and applied our main model and alternate frequency model to predict the probability that the target agent would interact with each other agent, i.e.:

[2]https://processing.org/

$P(\text{recip} = b|D, \text{init} = a)$. This yielded, for each possible trial, a vector of four probabilities for each model. We then computed the KL-divergence between the action distributions predicted by each model, and selected the 8 trials with the highest KL-divergences.

1.6.3 Experiment 3

For Experiment 3, we augmented each interaction sequence by randomly selecting 3 agents, and assigning each one a distinct choice type. We then applied our main model and frequency model to each sequence to predict, for each of the three agents/choice types, the amount of influence that each other agent would have over the target agent. This yielded, for each trial, target agent, and model, a vector of four values capturing the predicted degree of influence of each other agent. We then concatenated these three vectors of four values into a single vector of twelve values for each trial/agent/model. We then computed the sum-squared difference between each vector, and chose the 8 trials with the highest sum-squared difference.
