# Supplementary material for: Inferring the internal structure of groups through the integration of statistical learning and causal reasoning
Source: Nat Commun. 2026 Jan 23;17:1959. doi: 10.1038/s41467-026-68754-0 (PMC12929721; doi:10.1038/s41467-026-68754-0)
Supplement: Supplementary file 2 — Reporting Summary [file 41467_2026_68754_MOESM2_ESM.pdf]

nature portfolio

Corresponding author(s): Isaac Davis

Last updated by author(s): December 19, 2025

Reporting Summary

Nature Portfolio wishes to improve the reproducibility of the work that we publish. This form provides structure for consistency and transparency in reporting. For further information on Nature Portfolio policies, see our Editorial Policies and the Editorial Policy Checklist.

Statistics

For all statistical analyses, confirm that the following items are present in the figure legend, table legend, main text, or Methods section.

n/a	Confirmed	
☐	☒	The exact sample size (*n*) for each experimental group/condition, given as a discrete number and unit of measurement
☐	☒	A statement on whether measurements were taken from distinct samples or whether the same sample was measured repeatedly
☒	☐	The statistical test(s) used AND whether they are one- or two-sided<br>*Only common tests should be described solely by name; describe more complex techniques in the Methods section.*
☐	☒	A description of all covariates tested
☒	☐	A description of any assumptions or corrections, such as tests of normality and adjustment for multiple comparisons
☐	☒	A full description of the statistical parameters including central tendency (e.g. means) or other basic estimates (e.g. regression coefficient) AND variation (e.g. standard deviation) or associated estimates of uncertainty (e.g. confidence intervals)
☒	☐	For null hypothesis testing, the test statistic (e.g. *F*, *t*, *r*) with confidence intervals, effect sizes, degrees of freedom and *P* value noted<br>*Give P values as exact values whenever suitable.*
☐	☒	For Bayesian analysis, information on the choice of priors and Markov chain Monte Carlo settings
☒	☐	For hierarchical and complex designs, identification of the appropriate level for tests and full reporting of outcomes
☐	☒	Estimates of effect sizes (e.g. Cohen's *d*, Pearson's *r*), indicating how they were calculated

Our web collection on statistics for biologists contains articles on many of the points above.

Software and code

Policy information about availability of computer code

Data collection	Data collected via surveys implemented in Qualtrics and distributed via Prolific. Stimulus videos were generated in Processing (4.4.10) for macOS
Data analysis	Data analysis and visualization was performed using RStudio (version 2024.04.2+764) and R packages tidyverse (2.0.0), boot (1.3-28.1), and ggplot2 (3.5.2). Computational models were coded in WebPPL (v0.9.15). All analysis scripts and model code are available in an OSF repository at https://doi.org/10.17605/OSF.IO/M75DG

For manuscripts utilizing custom algorithms or software that are central to the research but not yet described in published literature, software must be made available to editors and reviewers. We strongly encourage code deposition in a community repository (e.g. GitHub). See the Nature Portfolio guidelines for submitting code & software for further information.

Data

Policy information about availability of data

All manuscripts must include a data availability statement. This statement should provide the following information, where applicable:

- Accession codes, unique identifiers, or web links for publicly available datasets
- A description of any restrictions on data availability
- For clinical datasets or third party data, please ensure that the statement adheres to our policy

All datasets generated and analyzed for the present studies are available in an OSF repository at: https://osf.io/m75dg/?view_only=5e5d1c3faa9d458da760ee9bc79d82c0

Research involving human participants, their data, or biological material

Policy information about studies with human participants or human data. See also policy information about sex, gender (identity/presentation), and sexual orientation and race, ethnicity and racism.

Reporting on sex and gender	Sample size reports provided with gender breakdown of participants per required policies (based on participants' self-reported gender identity), but data analysis and results were not split by gender: all reported results reflect all participants in sample, aggregating across gender identities
Reporting on race, ethnicity, or other socially relevant groupings	Mean and SD of participant ages were reported but not used in analysis in results. No other demographic factors recorded.
Population characteristics	Participants all had US-based IP addresses, were fluent English speakers, and had normal color vision. No other characteristics were used for filtering participants.
Recruitment	Participants were recruited online via Prolific
Ethics oversight	Yale Institutional Review board (protocol 200020357)

Note that full information on the approval of the study protocol must also be provided in the manuscript.

Field-specific reporting

Please select the one below that is the best fit for your research. If you are not sure, read the appropriate sections before making your selection.

☐ Life sciences ☒ Behavioural & social sciences ☐ Ecological, evolutionary & environmental sciences

For a reference copy of the document with all sections, see nature.com/documents/nr-reporting-summary-flat.pdf

Behavioural & social sciences study design

All studies must disclose on these points even when the disclosure is negative.

Study description	Quantitative experimental study, within-subject design
Research sample	We recruited participants through the online platform Prolific, as Prolific participants tend to be more attentive that participants on other online platforms. Participants were required to be at least 18 years of age, fluent English speakers, with US-based IP addresses and normal color vision The mean age was 37, and the total number of participants across all three experiments was 602, of whom 279 self-identified as women. The sample was not nationally representative.
Sampling strategy	No statistical methods were used to predetermine sample sizes, but our target sample sizes were pre-registered and similar to those reported in previous publications on social structure inference through online platforms
Data collection	Data collection done online, no in-person data collection. Participants were required to take the survey on computer (not tablet or phone) to ensure stimulus videos displayed correctly. Studies were within-subject, researcher aware of hypothesis during collection.
Timing	Experiment 1: 8/22/2023-8/24/2023. Experiment 2: 9/5/2023-9/8/2023. Experiment 3: 12/29/2023.
Data exclusions	Participants excluded for a) failing comprehension check question on both attempts or b) providing identical responses to all questions. Final sample/Total recruitment: Experiment 1: 251/433. Experiment 2: 264/379. Experiment 3: 87/130. Note that Experiments 1 & 2 each contained 3 sub-studies, each with a separate participant pool. Experiment 3 only contained one study.
Non-participation	No participants dropped out/declined to participate
Randomization	Each study was within-subject, so all participants completed all trials in each study. Trials were presented to participants in random order. Self-selection biases unlikely due to random recruitment through Prolific.

Reporting for specific materials, systems and methods

We require information from authors about some types of materials, experimental systems and methods used in many studies. Here, indicate whether each material, system or method listed is relevant to your study. If you are not sure if a list item applies to your research, read the appropriate section before selecting a response.

Materials & experimental systems

n/a	Involved in the study
☒	☐ Antibodies
☒	☐ Eukaryotic cell lines
☒	☐ Palaeontology and archaeology
☒	☐ Animals and other organisms
☒	☐ Clinical data
☒	☐ Dual use research of concern
☒	☐ Plants

Methods

n/a	Involved in the study
☒	☐ ChIP-seq
☒	☐ Flow cytometry
☒	☐ MRI-based neuroimaging

Plants

Seed stocks	*Report on the source of all seed stocks or other plant material used. If applicable, state the seed stock centre and catalogue number. If plant specimens were collected from the field, describe the collection location, date and sampling procedures.*
Novel plant genotypes	*Describe the methods by which all novel plant genotypes were produced. This includes those generated by transgenic approaches, gene editing, chemical/radiation-based mutagenesis and hybridization. For transgenic lines, describe the transformation method, the number of independent lines analyzed and the generation upon which experiments were performed. For gene-edited lines, describe the editor used, the endogenous sequence targeted for editing, the targeting guide RNA sequence (if applicable) and how the editor was applied.*
Authentication	*Describe any authentication procedures for each seed stock used or novel genotype generated. Describe any experiments used to assess the effect of a mutation and, where applicable, how potential secondary effects (e.g. second site T-DNA insertions, mosiacism, off-target gene editing) were examined.*
